# Myelomatous Pleural Effusion Revealing Underlying Plasma Cell Myeloma: An Uncommon Initial Presentation

**DOI:** 10.7759/cureus.91370

**Published:** 2025-08-31

**Authors:** Alexandra K Mawlong, Pranjal Kalita, Humsheer S Sethi, Vandana Raphael, Yasmeen Hynniewta, Sumanta Das, Naveen Kumar R, Biswajit Dey, Ashim Roy, Racheal S Marbaniang

**Affiliations:** 1 Pathology, North Eastern Indira Gandhi Regional Institute of Health and Medical Sciences, Shillong, IND; 2 Radiology, Shillong Medical College, Shillong, IND; 3 Radiology and Imaging, North Eastern Indira Gandhi Regional Institute of Health and Medical Sciences, Shillong, IND; 4 General Medicine, North Eastern Indira Gandhi Regional Institute of Health and Medical Sciences, Shillong, IND; 5 Biochemistry, North Eastern Indira Gandhi Regional Institute of Health and Medical Sciences, Shillong, IND

**Keywords:** m-spike, myelomatous pleural effusion, plasma cell, plasma cell myeloma, pleural effusion

## Abstract

Myelomatous pleural effusion (MPE), an uncommon manifestation of plasma cell myeloma (PCM), is characterized by proliferation of neoplastic monoclonal plasma cells (PCs). The incidence of MPE in patients of PCM is scarce and often portends an aggressive disease course. We report the case of a 55-year-old male patient who presented with progressive dyspnea for three days along with complaints of nonspecific back pain for three months. The presence of bilateral pleural effusion on chest radiography prompted a clinical suspicion of tuberculosis, leading to pleural fluid analysis. Pleural fluid analysis revealed an exudative effusion with elevated adenosine deaminase levels. Cytomorphology and cell block study of the pleural fluid revealed atypical PCs and plasma blasts. PC lineage and clonality were confirmed by CD138 positivity and kappa light chain restriction. Based on the cytomorphology findings, a diagnosis of MPE was suspected. A monoclonal "M" spike was noted in the serum protein electrophoresis study. Concomitant biochemical investigations revealed hypercalcemia, anemia, and deranged renal function. Subsequent skeletal radiography showed the presence of lytic bone lesions, and bone marrow examination confirmed PCM with 70% PCs. A diagnosis of PCM with MPE was established. Despite repeated counseling, the patient declined treatment and succumbed within one month of diagnosis. This case highlights the diagnostic significance of pleural fluid cytology in conjunction with clinical, radiological, and laboratory findings in identifying atypical presentations of PCM and also substantiates the poor prognosis associated with MPE. It is important to emphasize that early recognition and timely evaluation are crucial for patient management.

## Introduction

Plasma cell myeloma (PCM), formerly known as multiple myeloma, is primarily a bone marrow-based disorder characterized by multifocal proliferation of monoclonal neoplastic plasma cells (PCs). It is commonly associated with a monoclonal immunoglobulin (M protein) in serum and/or urine, along with evidence of organ damage. Bone marrow serves as the primary site of origin of this neoplasm for almost all cases [1]. PCM may be asymptomatic and diagnosed incidentally, or it may present as an aggressive neoplasm; hence, thorough clinical, radiological, and laboratory evaluation is of utmost importance. Bony pain due to lytic bone lesions, recurrent infections due to impaired humoral immunity, renal failure, and anemia are the classic symptoms of a patient with PCM. Other presentations may include bleeding and occasionally neurological manifestations due to spinal cord compression or peripheral neuropathy [2].

The incidence of nonmyelomatous pleural effusions (MPEs) in patients with multiple myeloma varies according to the medical literature. Pleural effusions as the initial presentation occurring as a direct consequence of infiltration of the pleura by neoplastic PCs, i.e., MPEs, are extremely rare (<1% of cases) and are associated with advanced disease and poor prognosis for the patient [3]. Here, we report an unusual case of PCM as pleural effusion being the initial presenting symptom in a 55-year-old male patient who presented to the emergency department complaining of shortness of breath, prompting a chest X-ray that revealed a bilateral pleural effusion.

## Case presentation

**Figure 1:**
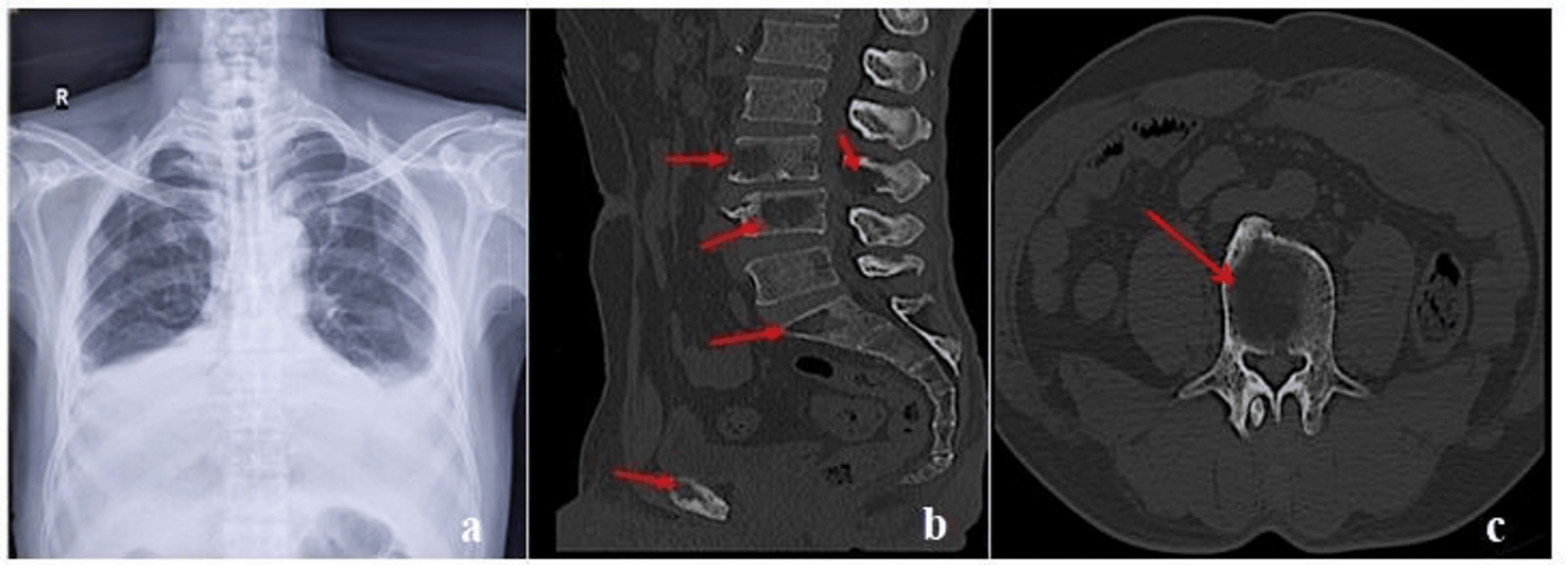
Chest X-ray showing evidence of bilateral pleural effusion. Sagittal CT of lumbar sacral spine in bone window (a). Multiple lytic lesions in the L4, L5, and S1 vertebral bodies, L3 spinous process, and superior pubic ramus (red solid arrow) (b). Axial CT of lumbar sacral spine in bone window showing lytic lesion in the L4 vertebral body (red solid arrow) (c) CT: computed tomography

A 55-year-old man presented to emergency services in a tertiary care institute with complaints of shortness of breath for three days, and on further questioning, he revealed that he had also been experiencing nonspecific back pain for three months. A chest X-ray revealed evidence of a bilateral pleural effusion, as shown in Figure 1a. Routine hematological and biochemical investigation was advised, along with pleural fluid analysis, and a clinical diagnosis of tuberculosis was suspected. No similar illness was reported in the family members. The patient also gave no history of any similar illness in the past. On examination, the patient was anemic, anicteric, and afebrile. Cardiovascular system and neurological examinations were within normal limits.

The pleural fluid analysis revealed an exudative pleural effusion with high adenosine deaminase levels. Elevated calcium levels and deranged kidney function were also noted. Laboratory investigation and relevant findings are listed in Tables 1, 2.

**Table 1:** Hematological findings in the patient TLC: total leucocyte count; DLC: differential leucocyte count

Investigations	Findings	Normal range
Complete blood count and peripheral blood smear analysis	Hemoglobin (g/dL) = 09.1	12-18 g/dL
	TLC (/uL) = 10.6 × 10^3^; DLC (%): neutrophil = 70; lymphocyte = 22; monocyte = 7; eosinophil = 1	TLC: 4-11 × 10^3^/uL. DLC (%): neutrophil: 40%-75%; lymphocyte: 20%-45%; monocyte: 2%-10%; eosinophils: 2%-6%
	Platelet count (/uL) = 450 × 10^3^	150-450/mL
	Peripheral blood smear study showed mild normocytic normochromic anemia and thrombocytosis. No atypical cells are noted in the smears studied	-
Erythrocyte sedimentation rate (mm/hour)	80	0-20 mm/hour
Packed cell volume (%)	33	36%-54%
Mean corpuscular volume (fL)	101	80-96 fL
Mean corpuscular hemoglobin (pg)	34	27-32 pg
Mean corpuscular hemoglobin concentration (%)	34	30%-36%
Prothrombin time (seconds)	10.6	9-12 seconds
Activated partial thromboplastin time (seconds)	22.7	21-36 seconds
International normalized ratio	0.990	0.8-1.1
D-dimer (mg/L)	<0.50	<0.50 mg/L
Fibrinogen (mg/dL)	70	180-300 mg/dL

**Table 2:** Biochemical and microbiological findings in the patient HBsAg: hepatitis B surface antigen; HCV: hepatitis C virus; HDL: high-density lipoprotein; LDL: low-density lipoprotein; VLDL: very low-density lipoprotein; HbA1c: hemoglobin A1c; TSH: thyroid-stimulating hormone; FT3: free triiodothyronine; PSA: prostate-specific antigen; CEA: carcinoembryonic antigen; PTH: parathyroid hormone; CBNAAT: Cartridge-Based Nucleic Acid Amplification Test; ADA: adenosine deaminase; GI: gastrointestinal; TLC: total leucocyte count; DLC: differential leucocyte count; TB: tuberculosis; CMD: corticomedullary differentiation

Biochemistry	On admission	On discharge	Normal range
Urea (mg/dL)	48	78	19.26-42.8 mg/dL
Creatinine (mg/dL)	2.5	3.8	0.66-1.25 mg/dL
Calcium (mg/dL)	10.72		8.5-10.2 mg/dL
Total bilirubin (mg/dL)	0.40	0.3	0.20-1.30 mg/dL
Indirect bilirubin	0.10	0.10	0.00-1.1 mg/dL
Aspartate aminotransferase (U/L)	28	31	17-59 U/L
Alanine transaminase (U/L)	14	20	4-50 U/L
Alkaline phosphatase (U/L)	60	66.3	38-126 U/L
Total protein (g/dL)	6.8	6.9	6.30-8.20 g/dL
Albumin (g/dL)	3.3	3.1	3.50-5.00 g/dL
Albumin/globulin ratio	0.9	0.8	1.25-1.51
HBsAg/anti-HCV/HIV	Negative/nonreactive/nonreactive		-
Amylase (U/L)	72		30-110 U/L
Lipase (U/L)	40		0-160 U/L
Cholesterol (mg/dL)	108		0-200 mg/dL
Triglycerides (mg/dL)	205		0-150 mg/dL
HDL (mg/dL)	21		40-60 mg/dL
LDL (mg/dL)	46		0-130 mg/dL
VLDL (mg/dL)	41		0-50 mg/dL
C-reactive protein (mg/L)	14.30		0-10 mg/L
Lactate dehydrogenase (U/L)	200		0-248 U/L
Uric acid (mg/dL)	4.4		2.6-7.2 mg/L
HbA1c	5.8%		<5.7%
TSH (mIU/L)	5.909		0.27-4.2 mIU/L
FT3 (ng/dL)	2.23		0.92-1.68 ng/dL
FT4 (pg/mL)	0.87		2.0-4.4 pg/mL
Vitamin B12 (pg/mL)	>1,500		191-663 pg/mL
Vitamin D (ng/mL)	28.27		11.1-42.9 ng/mL
Ferritin (ng/mL)	774.4		30-40 ng/mL
PSA (ng/mL)	0.133		0.01-1.4 ng/mL
CEA (ng/mL)	0.84		3.8-5 ng/mL
PTH (pmol/L)	1.22		0.16-6.9 pmol/L
Pleural fluid study	TLC: 80 cells/mm^3^. DLC: 90% monomorphs, 10% polymorphs. Atypical cells noted singly and in clusters		-
Pleural fluid culture	Sterile		-
Pleural fluid CBNAAT	Mycobacterium TB not detected		-
Pleural fluid (IU/L) adenosine deaminase (ADA)	154.10		-
Pleural fluid (IU/L) lactate dehydrogenase (LDH)	492		-
Pleural fluid (g/dL) protein	4.4		-
Ultrasonography (whole abdomen)	Hepatomegaly with Gr I hepatic steatosis. Bilateral increased renal cortical echotexture with diminished CMD. Acute on chronic renal parenchymal disease		-
Bronchoscopy	Normal study		-
Upper GI endoscopy	Normal study		-

**Figure 2:**
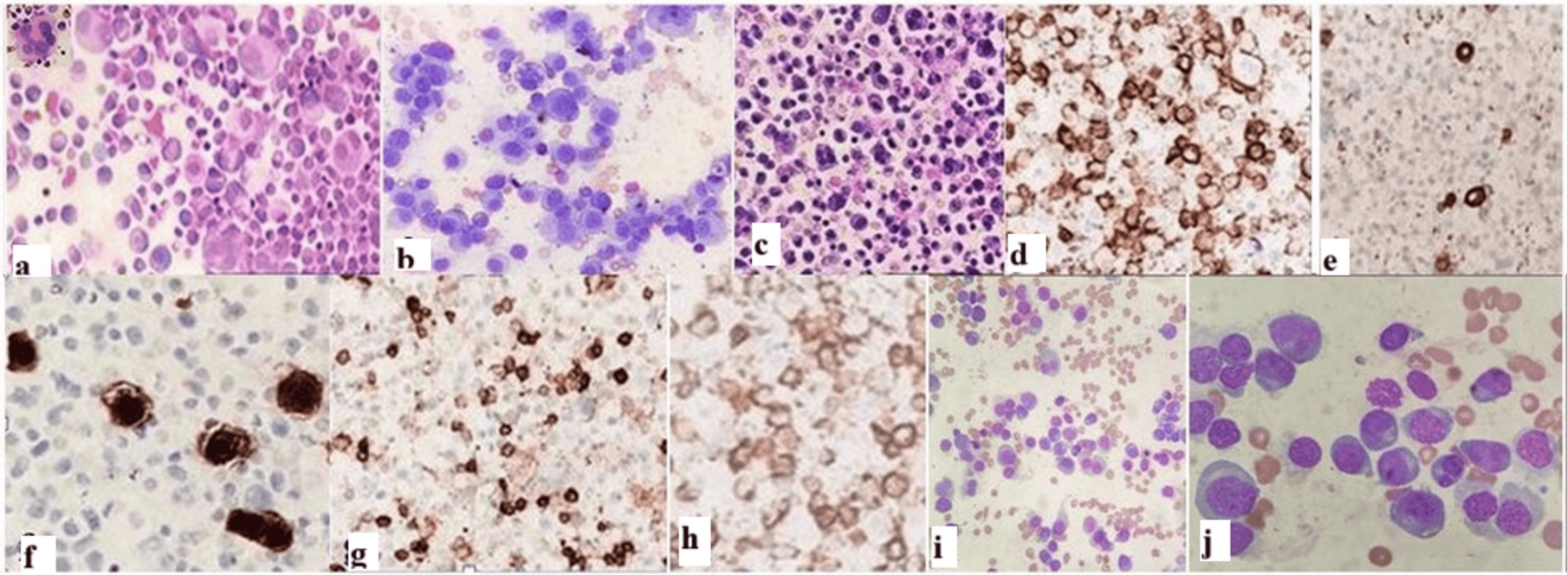
Pleural fluid analysis showing atypical plasma cells and plasmablasts (a,b H&E, MGG, 400x). Cell block analysis of pleural fluid showing atypical plasma cells and plasmablasts (c, H&E, 200x). Immunohistochemistry study showing positivity for CD138 (d, IHC, 400x). Pan CK, calretinin negative in neoplastic cells, positive in reactive mesothelial cells (e,f, IHC, 400x). LCA negative in neoplastic cells and positive in scattered lymphoid cells (g, IHC, 400x). Kappa immunostain positivity in neoplastic plasma cells (h, IHC, 400x). Bone marrow aspirate study showing abundant plasma cells with suppression of other hematopoietic cell lineages (i,j, Leishman stain, 100x, 400x) H&E: hematoxylin and eosin; MGG: May-Grünwald Giemsa; IHC: immunohistochemistry; LCA: leucocyte common antigen

Cytomorphological examination of the pleural fluid revealed a hypercellular smear composed of abundant atypical PCs and plasmablasts admixed with reactive mesothelial cells and scattered lymphoid cells. Cellblock analysis was carried out for the patient, revealing a similar morphology to the smears examined. Cluster of differentiation 138 (CD 138) positivity, aided by cytomorphology, along with negativity for markers like pancytokeratin (Pan CK), calretinin, and leucocyte common antigen (LCA) in neoplastic cells, confirmed the PC lineage of the atypical cells. Pan CK and calretinin positivity highlighted the reactive mesothelial cells, while LCA was positive in the scattered reactive lymphoid population. The neoplastic PCs were monoclonal, expressing Kappa immunoglobulin light chain, but showed no lambda expression, as shown in Figures 2a-2h. Serum protein electrophoresis (SPEP) was performed, revealing hypoalbuminemia with decreased beta 1, beta-2 globulin variants, and hypergammaglobulinemia. A monoclonal "M" spike was noted in the gamma region. SPEP findings and summary are highlighted in Table 3.

**Table 3:** Protein fractions and concentration in serum protein electrophoresis

Protein fractions	%	Reference (%)	Concentration (g/dL)	Reference concentration (g/dL)
Albumin	43.8	55.8-66.1	3	4-4.8
Alpha 1	4.7	2.9-4.9	0.3	0.2-0.4
Alpha 2	10.2	7.1-11.8	0.7	0.5-0.9
Beta 1	4.1	4.7-7.2	0.3	0.3-0.5
Beta 2	2.5	3.2-6.5	0.2	0.2-0.5
Gamma	34.7	11.1-18.8	2.4	0.8-1.4

The findings thus prompted a probable diagnosis of MPE. Based on the pleural effusion findings correlating with relevant laboratory findings, and considering the patient's complaint of low backache, a computed tomography was done, which showed multiple lytic bony lesions, as shown in Figures 1b, 1c. The patient was managed conservatively and advised to undergo a bone marrow examination. Bone marrow aspirate showed increased atypical PCs and plasmablasts (70%) with suppression of other hematopoietic cell lineages, as shown in Figures 2i, 2j. Bone marrow biopsy showed nodules and a sheet of atypical PCs. The bone marrow aspiration and biopsy confirmed the diagnosis of PCM. A final diagnosis of PCM with MPE was suggested. No cytogenetic study was carried out, considering financial and resource constraints. The Revised International Staging System (R-ISS) is the preferred staging system and has replaced the previous systems used for staging. As no cytogenetic study was done, the R-ISS system was not used. As per the international staging system (ISS) for myeloma, the patient was Stage III (serum albumin levels: 3.3 g/dL, and beta-2 microglobulin: 5.8 mg/L). The patient refused further treatment and left against medical advice. The patient was counseled about the treatment protocols and advised to consider treatment; however, the patient refused and succumbed to his illness one month after diagnosis.

## Discussion

Extramedullary disease (EMD) portends advanced stages of PCM and predicts poor survival. The incidence of EMD appears to be increasing, which may be attributed to the greater availability and sensitivity of diagnostic tests, as well as advancements in therapeutics, resulting in improved patient survival [4]. In the majority of patients with PCM, PC proliferation is confined to the bone marrow; however, extramedullary involvement, albeit rare, has been reported in the medical literature. These clonal PCs exhibit the ability to thrive and grow independently of the bone marrow microenvironment, representing an aggressive form of PCM characterized by increased proliferation, high-risk genetic features, evasion of apoptosis, and resistance to therapy. Typical sites of EMD may vary according to the stage of PCM. At the time of diagnosis, EMD is typically found in the skin; during relapse, sites commonly involved include the liver, kidneys, lymph nodes, breast, pleura, pericardium, and the central nervous system [5]. Involvement of the serous cavities is uncommon.

The etiology of pleural effusion in PCM may be varied, ranging from congestive heart failure, pneumonia, tuberculosis, carcinomatosis, hypoproteinemia, and other viral illnesses. With disease progression, approximately 6% of PCM patients develop pleural effusions, but fewer than 1% of patients present as MPE [6]. Studies by Awasthi et al. and Cakir et al. reported three and two patients with MPE among 164 and 364 malignant pleural effusions analyzed, respectively [7,8]. In a study by Wang et al., which included 3,480 patients with pleural effusion and 319 patients with PCM, there were 34 patients with both conditions (17 men and 17 women). However, there were only two MPE cases [9]. The patient in discussion in our case report had normal cardiac function and no evidence of infection, pulmonary embolism, or nephrotic syndrome. There was no evidence of secondary malignancies. Furthermore, MPE presents most commonly as unilateral effusions [10]. To the best of our knowledge, only 11 cases of bilateral MPE have been reported in the literature. The gold standard for diagnosing MPE is cytopathologic evidence of PC infiltration in the pleural fluid.

However, the anaplastic nature of the extramedullary PCs poses a diagnostic challenge. In our reported case, MPE was diagnosed cytopathologically in conjunction with SPEP of the pleural fluid, revealing a monoclonal "M" spike with raised calcium level, deranged kidney function, presence of anemia, and lytic bone lesions radiologically. The overall median survival time for MPE is four months (ranging from 3 to 50 months), prompting a detailed systematic approach to exclude alternative diagnoses [11]. This is our first experience of a patient presenting with a bilateral pleural effusion as an initial symptom of PCM. In the reported case, the patient succumbed to his illness one month after diagnosis, which indicates the aggressive nature and dismal prognosis.

Cytogenetic evaluation plays a key role in multiple myeloma (MM) patients. Significant role of cytogenic evaluation in MM can be ascertained by the fact that cytogenetic findings are incorporated in the staging and its eventual role in prognosis, and response to therapy, as mentioned in a study by Abdallah et al. [12]. In our reported case, due to financial and resource constraints, cytogenetic studies were not conducted. However, the authors opine that cytogenetic evaluation is of immense importance in cases of MM and especially in aggressive cases, as response to therapy and eventual prognosis depend on it.

## Conclusions

The case report demonstrates the importance of considering MPE as a possible initial presentation of PCM. Suspecting malignant effusions is of utmost importance as it portends a poorer prognosis; furthermore, in any hematologic malignancies, it is essential to evaluate the etiology of pleural effusion as the treatment guidelines and prognostic implications differ widely for each etiology. Despite vast advancements in the therapeutic strategies of PCM, the median survival in MPE is poor, given the aggressive symptomatic course and short survival.
